# Gata3 is required for the functional maturation of inner hair cells and their innervation in the mouse cochlea

**DOI:** 10.1113/JP277997

**Published:** 2019-05-28

**Authors:** Tanaya Bardhan, Jing‐Yi Jeng, Marco Waldmann, Federico Ceriani, Stuart L. Johnson, Jennifer Olt, Lukas Rüttiger, Walter Marcotti, Matthew C. Holley

**Affiliations:** ^1^ Department of Biomedical Science University of Sheffield Sheffield UK; ^2^ Department of Otolaryngology Tübingen Hearing Research Center Section of Physiological Acoustics and Communication University of Tübingen 72076 Tübingen Germany

**Keywords:** Hypoprathyroidism, Deafness and Real anomaly Syndrome, cochlea, Hair cells, Gata3, Development, afferent fibre, Deafness

## Abstract

**Key points:**

- The physiological maturation of auditory hair cells and their innervation requires precise temporal and spatial control of cell differentiation.
- The transcription factor *gata3* is essential for the earliest stages of auditory system development and for survival and synaptogenesis in auditory sensory afferent neurons.
- We show that during postnatal development in the mouse inner ear gata3 is required for the biophysical maturation, growth and innervation of inner hair cells; in contrast, it is required only for the survival of outer hair cells.
- Loss of gata3 in inner hair cells causes progressive hearing loss and accounts for at least some of the deafness associated with the human hypoparathyroidism, deafness and renal anomaly (HDR) syndrome.
- The results show that gata3 is critical for later stages of mammalian auditory system development where it plays distinct, complementary roles in the coordinated maturation of sensory hair cells and their innervation.

**Abstract:**

The zinc finger transcription factor gata3 regulates inner ear development from the formation of the embryonic otic placode. Throughout development, *gata3* is expressed dynamically in all the major cochlear cell types. Its role in afferent formation is well established but its possible involvement in hair cell maturation remains unknown. Here, we find that in heterozygous *gata3* null mice (*gata3^+/−^*) outer hair cells (OHCs) differentiate normally but their numbers are significantly lower. In contrast, inner hair cells (IHCs) survive normally but they fail to acquire adult basolateral membrane currents, retain pre‐hearing current and efferent innervation profiles and have fewer ribbon synapses. Targeted deletion of *gata3* driven by otoferlin‐cre recombinase (*gata3^fl/fl^otof‐cre^+/−^*) in IHCs does not affect OHCs or the number of IHC afferent synapses but it leads to a failure in IHC maturation comparable to that observed in *gata3^+/−^* mice. Auditory brainstem responses in *gata3^fl/fl^otof‐cre^+/−^* mice reveal progressive hearing loss that becomes profound by 6–7 months, whilst distortion product otoacoustic emissions are no different to control animals up to this age. Our results, alongside existing data, indicate that gata3 has specific, complementary functions in different cell types during inner ear development and that its continued expression in the sensory epithelium orchestrates critical aspects of physiological development and neural connectivity. Furthermore, our work indicates that hearing loss in human hypoparathyroidism, deafness and renal anomaly (HDR) syndrome arises from functional deficits in IHCs as well as loss of function from OHCs and both afferent and efferent neurons.

## Key points

- The physiological maturation of auditory hair cells and their innervation requires precise temporal and spatial control of cell differentiation.
- The transcription factor *gata3* is essential for the earliest stages of auditory system development and for survival and synaptogenesis in auditory sensory afferent neurons.
- We show that during postnatal development in the mouse inner ear gata3 is required for the biophysical maturation, growth and innervation of inner hair cells; in contrast, it is required only for the survival of outer hair cells.
- Loss of gata3 in inner hair cells causes progressive hearing loss and accounts for at least some of the deafness associated with the human hypoparathyroidism, deafness and renal anomaly (HDR) syndrome.
- The results show that gata3 is critical for later stages of mammalian auditory system development where it plays distinct, complementary roles in the coordinated maturation of sensory hair cells and their innervation.

## Introduction

The auditory system is composed of highly organised arrays of inner hair cells (IHCs), outer hair cells (OHCs) and surrounding non‐sensory cells (Whitfield, 2015; Basch *et al*. 2016). The progression and timing of cell differentiation and innervation are critical for the formation of the tonotopic map and for the development of complementary structures, for example, the pre‐ and postsynaptic structures in IHCs and their sensory neurons (Sobkowicz *et al*. 1986; Yu *et al*. 2013). Early physiological activity is an essential part of development. Hair cell functional differentiation depends upon Ca^2+^‐dependent action potentials during discrete periods of pre‐hearing development (IHCs: Johnson *et al*. 2013; OHCs: Ceriani *et al*. 2019) as well as on the acquisition of mechano‐electrical transducer currents (Corns *et al*. 2018), which are in turn driven by the developing endocochlear potential (Bosher & Warren, 1971). Regulation of the timing and interplay between genetic programming and cell physiology is thus crucial for inner ear development.

The zinc finger transcription factor, gata3, is a key regulator of auditory system development (Karis *et al*. 2001; Milo *et al*. 2009; Appler *et al*. 2013). It is expressed in most embryonic tissues (Nardelli *et al*. 1999; Hendricks *et al*. 1999; Kurek *et al*. 2007; Nishiyama *et al*. 2009; Zhang *et al*. 2007; Boualia *et al*. 2013) and in almost all auditory cell types (Rivolta & Holley, 1998; Lawoko *et al*. 2002; Milo *et al*. 2009). Its dynamic regulation suggests that it orchestrates cell differentiation as the auditory system develops (Milo *et al*. 2009; Appler *et al*. 2013). Furthermore, haploinsufficiency for gata3 leads to hypoparathyroidism, deafness and renal anomaly (HDR) syndrome (Van Esch *et al*. 2000; Martins *et al*. 2018). Heterozygous null *gata3* mice (*gata^+/−^*) have a complex cochlear pathology, including OHC degeneration as early as 1 month of age, followed by loss of pillar cells, IHCs and nerve fibres (van der Wees *et al*. 2004). Auditory brainstem responses (ABRs) show that *gata^+/−^* mice have a 30 dB hearing loss from the normal onset of hearing, believed to be caused by OHC loss (van der Wees *et al*. 2004; Van Looij *et al*. 2005).

Gata3 is expressed through all stages of development of the auditory sensory apparatus, although the highest level occurs before birth and declines towards the onset of hearing (Milo *et al*. 2009). *Gata3* null mice can be maintained until about embryonic day E16.5 but their cochleas remain stunted and the only hair cells that form are found in the vestibular system (Lim *et al*. 2000; Duncan *et al*. 2011; Haugas *et al*. 2012; Luo *et al*. 2013). Cochlea underdevelopment occurs when *gata3* is deleted from around E8.5 using a foxg1‐Cre driver and a less severe phenotype is found with a Pax2‐Cre driver, which deletes *gata3* from around E10 (Duncan & Fritzsch 2013). Direct targets of *gata3* that are critical for inner ear development include fgf10 (Lillevali *et al*. 2006) and potentially cyclinD1 (Molenaar *et al*. 2010), which is downregulated selectively in hair cells as they differentiate (Laine *et al*. 2010), following the pattern for gata3 (Rivolta & Holley 1998). However, the function of gata3 may differ between cell types at different stages of development, as illustrated for OHCs (van der Wees *et al*. 2004) and SGNs (Yu *et al*. 2013).

In this paper we explore the function of gata3 in cochlear hair cells. We found that haploinsufficiency and deletion of *gata3* have different effects in IHCs and OHCs. The data provide insight into the function of *gata3* in the orchestration of differentiation of different cochlear cell types during the later stages of development.

## Methods

### Ethics statement

All animal work performed at the University of Sheffield (UK) was licensed by the Home Office under the Animals (Scientific Procedures) Act 1986 and was approved by the University of Sheffield Ethical Review Committee (approval reference no. PCC8E5E93). In Germany, animal experiments were performed according to the European Union Directive 2010/63/EU for the protection of animals used for experimental and other scientific purposes and approved by the animal welfare commissioner and the regional board for animal experimentation.

### Transgenic mouse lines

*Gata3* homozygous null mice die at early embryonic stages (E10.5–E13.5: Pandolfi *et al*. 1995; Karis *et al*. 2001). We studied the effects of *gata3* haploinsufficiency in heterozygous null mice in which one *gata3* allele was replaced with a *taulacZ* reporter gene (Hendriks *et al*. 1999; van der Wees *et al*. 2004). To target *gata3* deletion to hair cells we crossed a floxed *gata3* strain, in which exon 4 of the *gata3* gene is flanked by *loxP* sites (Zhu *et al*. 2004) with *otoferlin*‐cre mice (Kazmierczak *et al*. 2017), which express cre‐recombinase under the otoferlin promoter. In the cochlea, otoferlin is expressed exclusively in hair cells, including some 90% of the IHCs and 10% of the OHCs. Genotyping for the conditional mice was performed by PCR with the following primers: of *gata3* 5ʹ‐TCAGGGCACTAAGGGTTGTTAACTT‐3ʹ; 5ʹ‐GAATTCCATCCATGAGACACACAA‐3ʹ; *otof‐cre* 5ʹ CAGCACACTGGCGGCCGTTACTA 3ʹ; 5ʹ AGAGAAACACAAGGTCGGGCTCAATCT 3ʹ; 5ʹ TGGTCGGGTCTGTGGTGTTACAACT 3ʹ. Throughout this report wild‐type, heterozygous null and homozygous null mice are referred to as *gata3^+/+^*, *gata3^+/−^* and *gata3^−/−^* mice, respectively. Heterozygous and homozygous *gata3* floxed mice are referred to as *gata3^+/fl^ or gata3^fl/fl^*, respectively and heterozygous and homozygous cre‐recombinase mice are *otof‐cre^+/−^* and *otof‐cre^−/−^*, respectively.

### Tissue preparation

IHCs and OHCs were studied in acutely dissected, postnatal organs of Corti, where the day of birth was postnatal day P0. Animals of either sex were killed by cervical dislocation and the organs of Corti dissected in extracellular solution composed of (in mM): 135 NaCl, 5.8 KCl, 1.3 CaCl_2_, 0.9 MgCl_2_, 0.7 NaH_2_PO_4_, 5.6 d‐glucose, 10 HEPES‐NaOH. Sodium pyruvate (2 mM), amino acids and vitamins were added from concentrates (Fisher Scientific, Loughborough, UK). The pH was adjusted to 7.5 (osmolality ∼308 mmol kg^−1^). The dissected apical coil of the organ of Corti was transferred to a microscope chamber, immobilized using a nylon mesh fixed to a stainless steel ring and viewed using an upright microscope (Leica DMLFS, Milton Keynes, UK; Nikon FN1, Kingston upon Thames, UK). Hair cells were observed with Nomarski differential interface contrast optics (63× water immersion objectives). To expose the basolateral surface of the cells, a small tear was made in the epithelium with a suction pipette (tip diameter 3–4 μm) filled with extracellular solution.

### Single‐cell electrophysiology

Hair cell membrane currents and voltage responses were investigated under whole cell voltage or current clamp, respectively, at room temperature (20–25°C), using an Optopatch amplifier (Cairn Research Ltd, Faversham, UK). Patch pipettes were pulled from soda glass capillaries (Harvard Apparatus Ltd, Edenbridge, UK) and their shanks coated with surf wax (Mr Zoggs Sex Wax, CA, USA) to reduce the electrode capacitative transient. The intracellular solution was composed of (mM): 131 KCl, 3 MgCl_2_, 1 EGTA‐KOH, 5 Na_2_ATP, 5 HEPES‐KOH, 10 sodium phosphocreatine (pH 7.28, 294 mmol kg^−1^). Data was filtered at 2.5 kHz (8‐pole Bessel) and sampled at 5 kHz. Origin 2018 software (OriginLab, Northampton, MA, USA) was used to perform offline data analysis. Membrane potentials were corrected for the residual series resistance *R*_s_ after compensation (usually 80%) and liquid junction potential (LJP), which was −4 mV. When investigating basolateral membrane properties, the size of *I*_K,f_ was measured near –25 mV and at 2.0 ms after the start of the voltage step, while *I*_K,n_ was measured as the difference between the peak and steady state of the deactivating inward current at −124 mV. Steady‐state total currents were measured at 160 ms, at a potential of 0 mV (extrapolated from the current‐voltage curves). The holding potentials used for these recordings were set at either −84 mV or −64 mV (normally specified in the figure legends).

### Statistical analysis for the single cell physiology

Mean values were compared with Student's two‐tailed *t* test or, for multiple comparisons, one‐way and two‐way analysis of variance (ANOVA) followed by Bonferroni's test). *P* < 0.05 was selected as the criterion for statistical significance. Mean values are quoted in text and figures as means ± SEM. Those referring to *in vivo* physiology measurements (ABRs and DPOAEs) are reported as means ± SD. Animals of either sex were randomly assigned to the different experimental groups. No statistical methods were used to define sample size, which was based on previously published, similar work from our laboratory. The majority of the experiments were performed blind with respect to animal genotyping.

### Cochlear culture preparation

Cochlear cultures from postnatal mice were prepared as described previously (Richardson & Russell, 1991; Corns *et al*. 2017). Briefly, cochleae were dissected in HEPES buffered (10 mm, pH 7.2) Hanks’ balanced salt solution (HBHBSS), placed onto collagen‐coated glass coverslips, fed with 100–150 μl of medium containing 98% or 93% standard Dulbecco's modified Eagle's DMEM/F12 with additional 10 mm HEPES buffer, 2% or 7% fetal bovine serum (Labtech International Limited, Uckfield, UK) and 10 μg/ml ampicillin (Sigma‐Aldrich, Gillingham, UK) and maintained at 37°C for 24 h. Then, cochlear cultures were fixed with 4% paraformaldehyde in phosphate buffered saline (PBS) for 1 h at 22°C, washed three times with PBS and stained with a solution containing Alexa Fluor 488 phalloidin (1:300: Life Technologies Ltd, Paisley, UK; RRID:AB_2315147), 0.7% FBS and 0.01% Triton ‐X100 for 2 h. The coverslips were then washed another three times in PBS and the collagen with the attached cochleae was peeled off from the coverslips and mounted in Vectashield mounting medium (Vector Laboratories, Peterborough, UK; RRID:AB_2336789). Cochleae were imaged with an Olympus BXB61 with 10× or 20× dry objectives and images were captured using the Volocity 3D Image Analysis Software (RRID:SCR_002668). The number of hair cells along the four different cochlear regions (see Results section) was measured over a 150 mm length region using Photoshop.

### Immunofluorescence microscopy

In the UK, dissected cochleae (≥3 mice for each set of experiment) were fixed with 4% paraformaldehyde in phosphate‐buffered saline (PBS, pH 7.4) for 5–20 min at room temperature. Cochleae were microdissected, rinsed three times for 10 min in PBS and incubated for 1 h at room temperature in PBS supplemented with 5% normal goat or horse serum and 0.3% Triton X‐100. The samples were then incubated overnight at 37˚C with the primary antibody in PBS supplemented with 1% of the specific serum. Primary antibodies were: mouse anti‐myosin7a (1:100, DSHB, Iowa City, IA, USA; no. 138‐1S), rabbit anti‐myosin7a (1:500, Proteus Biosciences, Nottingham, UK; no. 25‐6790), mouse anti‐CtBP2 (1:200, BD Biosciences, Berkshire, UK; no. 612044), rabbit anti‐SK2 (1:500, Sigma‐Aldrich, no. P0483), goat anti‐choline acetyltransferase (ChAT, 1:500, Millipore, Hertfordshire, UK; no. AB144P) and mouse anti‐PSD95 (1:1000, Millipore, no. MABN68). All primary antibodies were labelled with species‐appropriate Alexa Fluor secondary antibody for 1 h at 37˚C. Samples were then mounted in Vectashield. The *z*‐stack images were captured with the GaAsP detectors or with a Nikon A1 confocal microscope. Image stacks were processed with Fiji Image Analysis software. Mean values were compared with Student's two‐tailed *t* test with *P* < 0.05 selected as the criterion for statistical significance.

### Hearing measurements

For hearing measurements animals were anaesthetized with a mixture of fentanyl (0.05 mg kg^−1^ body weight), Dormicum (midazolam, 5 mg kg^−1^ body weight) and medetomidine (medetomidine hydrochloride, Dormitor, 0.5 mg kg^−1^ body weight), injected i.p. Additional doses of anaesthetics were administered if needed and body temperature was maintained by heating pads and lamps.

ABRs and DPOAEs were measured in a soundproof chamber (IAC 400‐A, Industrial Acoustics Company GmbH, Niederkrüchten, Germany) as previously described (e.g. Zuccotti *et al*. 2012). Briefly, ABRs and the cubic 2^*^f1‐f2 DPOAE for f2 = 1.24^*^f1 were recorded in anaesthetized adult animals. Electrical brainstem responses to free field click (100 μs, 0–100 dB SPL), noise burst (1 ms random phase) and pure tone (2–45.25 kHz in half‐octave steps, 3 ms, 1 ms cosine squared rise‐fall envelope) acoustic stimuli were recorded with subdermal silver wire electrodes at the ear (positive, active), the vertex (negative, reference) and the back of the animals (ground). Recordings were made for 10 ms with stimulus presentations of alternating polarity to eliminate electrical artefacts. In each case, stimulus presentation started at time 0 ms. The click stimulus was a broadband stimulus with a centre frequency at 4.9 kHz (50th percentile) and the 25th and 75th percentiles at 2.2 kHz and 13.8 kHz, respectively. Signals were amplified (100K‐fold), bandpass filtered (0.2–5 kHz 6‐pole Butterworth filter, Wulf Elektronik, Frankfurt, Germany), averaged across 64–256 repetitions (dependent on the signal to noise ratio, but always the maximal repetition number at close threshold stimulation) at each sound pressure presented (usually 0–100 dB SPL in steps of 5 dB) and recorded at 20 kHz sample frequency. Stimuli were delivered to the ear in a calibrated open system by a loudspeaker (DT‐911, Beyerdynamic, Heilbronn, Germany) placed 3 cm lateral to the animal's pinna. Sound pressure was calibrated online prior to each measurement with a microphone (B&K 4135, Bruel & Kjaer, Naerum, Denmark) placed near the animal's ear.

For stimulus generation and signal recording of ABRs, a multi‐function IO‐Card (PCI‐6052E, National Instruments, Austin, TX, USA) was used, housed in an IBM compatible computer. Sound pressure level was controlled with an attenuator and amplifier (Wulf Elektronik). To reduce physical stress of the animals by long lasting anaesthesia to a minimum, ABR measurement times were reduced to a minimum by increasing stimulus repetition rates to 62 s^−1^, minimizing repetition numbers for clearly suprathreshold signals (when ABR wave amplitudes were exceeding ±4 μV).

#### ABR analysis

Hearing threshold was determined by the lowest sound pressure that produced visually distinct evoked potentials from above threshold to near threshold.

#### Peak input‐output (I/O) analysis

For each individual ear the ABR wave data for the click and noise stimuli were analysed for peak, trough amplitudes and the latencies by customized computer programs. From individual ABR waves to click and noise stimuli, peak amplitudes and peak latencies were collected, grouped in clusters of similar peak amplitude and latencies and averaged for ABR wave input‐output (I/O) analysis. ABR wave amplitudes were defined as peak to peak amplitude of a negative peak (n) followed by a positive (p) peak. For selected peaks and troughs the I/O functions were derived from the peak‐to‐peak amplitudes at all recorded stimulus sound pressure levels. Two peak classes were selected: (1) early peaks (at 0.85–1.65 ms (‘wave Ia’) and (2) delayed peaks at 3.6–5.9 ms (‘wave IV’).

#### DPOAE analysis

DPOAE growth (I/O) functions in age groups 1.7–2.3 months and older than 7 months were inspected for errors from incorrectly placed loudspeakers. Only animals with an averaged DPOAE between 10 and 30 dB SPL within the confidence interval of the five highest averaged DPOAE between 10 and 30 dB SPL were included for the analysis.

### Statistical analysis of hearing measurements

Data are presented as means ± standard deviation (SD). Differences of the mean were compared for statistical significance either by one‐way, or two‐way ANOVA and Bonferroni or Turkey tests as *post hoc* tests (Graphpad Prism version 6.01). For wave I and IV amplitude slopes (Figs 9 and 10) mean data was compared with two‐way ANOVA (single gaps in the dataset were interpolated by weighted running average). When two‐way ANOVA could not be used, data were compared with Pearson's χ^2^ and Fisher exact probability tests (VassarStats, 2018). ^*^ *P* < 0.05; ^**^ *P* < 0.01; ^***^ *P* < 0.001; ^****^ *P* < 0.0001; n.s., not significant.

## Results

**Figure 1:**
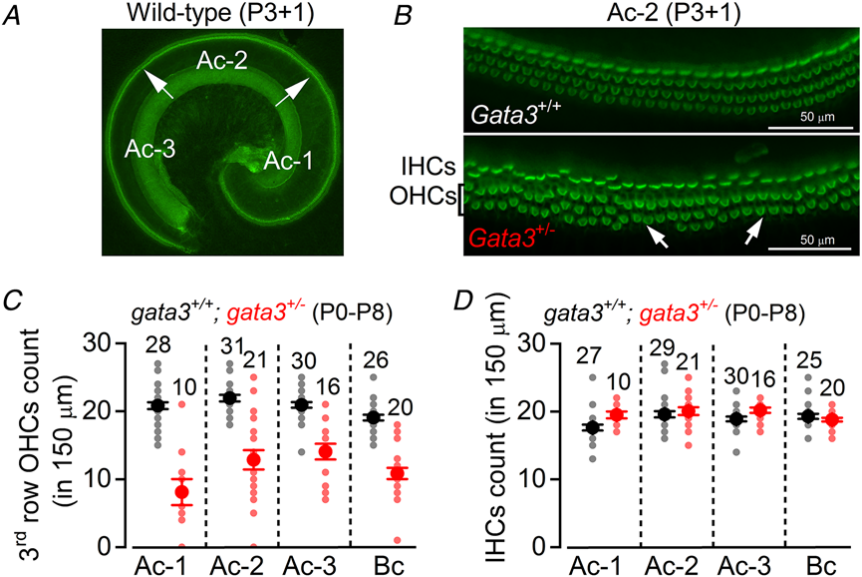
OHCs from *gata3^+/^^−^* mice are reduced in number *A*, fluorescence images from the apical coil of the cochlea of P3 control *gata3^+/+^* mice taken after incubation of the tissue with Alexa Fluor 488 phalloidin. The arrows indicate the position of the hair cells along the apical coil from apical (Ac‐1) to middle (Ac‐2) and base (Ac‐3). *B*, expanded images from the region Ac‐2 (see panel *A*) for wild‐type (top) and *gata3^+/−^* (bottom) mice where the hair bundle of both IHCs (one row) and OHCs (three rows) are visible. Note that some OHCs in the 3rd row are missing in the *gata3^+/−^* mouse cochlea (arrows). *C* and *D*, number of 3rd row OHCs (*C*) and IHCs (*D*) present in a 150 μm length of the sensory epithelium from the three different apical positions highlighted in panel *A*, plus one basal coil region (Bc).

*Gata3^+/−^* mice have a 30 dB permanent threshold shift in their hearing from 1 month of age and early morphological degeneration of OHCs (van der Wees *et al*. 2004). Alongside a deterioration of distortion product otoacoustic emissions (DPOAEs), this suggests that loss of OHCs is likely to be responsible for hearing loss in the *gata3^+/−^* mouse and a consequence of *gata3* haploinsufficiency in HDR syndrome (van Looij *et al*. 2005). Consistent with this view, our data showed that in *gata3^+/−^* mice the number of OHCs in the 3rd or most external row (Fig. 1 *A–C*), but not the 1st and 2nd rows, was already significantly reduced (two‐way ANOVA, *P* < 0.0001) during pre‐hearing stages compared to that of wild‐type *gata3^+/+^* littermates (Fig. 1 *C*). IHC numbers were similar in the two genotypes (Fig. 1 *D*). We then investigated the biophysical properties of both hair cell types to look for the earliest signs of functional decline.

### The biophysical properties of mature IHCs, but not OHCs, are affected in *gata3^+/−^* mice

**Figure 2:**
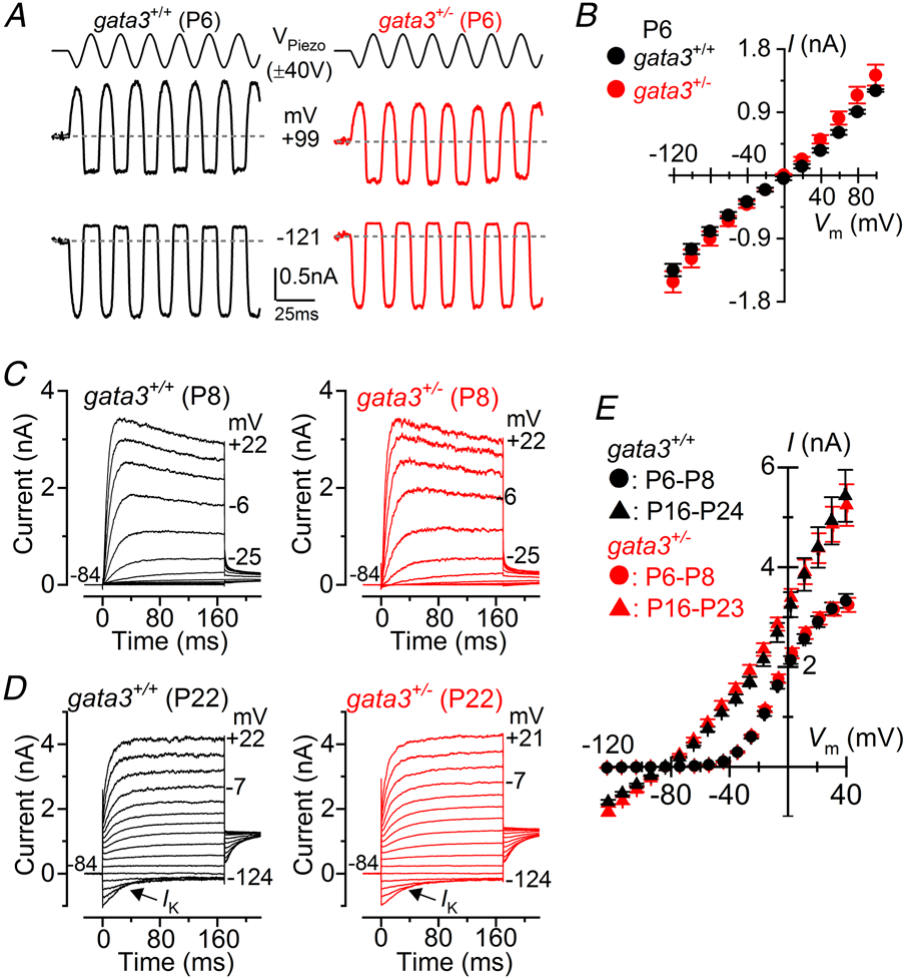
The remaining OHCs from *gata3^+/^^−^* mice become functionally mature *A*, saturating MET currents in P6 apical OHCs from *gata3* ^+/+^ (left) and *gata3^+/−^* (right) mice in response to a 50 Hz sinusoidal force stimulus to the hair bundles at the membrane potentials of −121 mV and +99 mV. Dashed lines indicate the holding current. *V*_Piezo_ indicates the driver voltage to the fluid jet, with positive deflections moving the hair bundles in the excitatory direction. *B*, peak‐to‐peak MET current‐voltage curves obtained from 5 *gata3* ^+/+^ and 3 *gata3^+/−^* OHCs at P6. Recordings were obtained using the same protocol shown in panel *A*, but using voltage steps in 20 mV nominal increments from −121 mV to +99 mV. *C*, K^+^ currents elicited from P8 OHCs (control: black; mutant: red) by applying depolarizing and hyperpolarizing voltage steps in 10 mV nominal increments, starting from the holding potential of –84 mV. *D*, K^+^ currents recorded from mature P22 control (black) and mutant (red) OHCs elicited by depolarizing voltage steps (10 mV nominal increments) from –124 mV to more depolarized values from the holding potential of –84 mV. *E*, peak current‐voltage curves obtained from OHCs of *gata3^+/+^* (P6–P8: *n* = 18; P16–P24: *n* = 12) and *gata3^+/−^* (P6–P8: *n* = 34; P16–P23: *n* = 7) mice. In this and all other figures single‐cell recordings were performed at room temperature.

Saturating mechanoelectrical transducer (MET) currents from apical coil OHCs of *gata3^+/+^* and littermate *gata3^+/−^* mice were elicited by displacing their hair bundles with sinewave stimuli from a piezoelectric fluid jet stimulator (Corns *et al*. 2014, 2016; Marcotti *et al*. 2016). At −121 mV, bundle displacement in the excitatory direction (i.e., towards the taller stereocilia) elicited large inward MET currents from postnatal day 6 (P6) OHCs of both genotypes (Fig. 2 *A*). Membrane depolarization (+99 mV) caused the MET current to decrease in size at first and then reverse near 0 mV to become outward at positive potentials (Fig. 2 *A*). The maximum amplitude of the MET current was similar between the two genotypes (Fig. 2 *B*).

When we investigated the basolateral membrane currents we found that steady‐state outward K^+^ currents in immature OHCs were indistinguishable between the two genotypes (*I*_K_: *gata3^+/+^* 2.01 ± 0.07 pA, *n* = 18; *gata3^+/−^* 2.11 ± 0.10 pA, *n* = 34, measured at 0 mV; *P = *0.4835: Fig. 2 *C* and *D*). Mutant OHCs also expressed a K^+^ current called *I*_K,n_ (Fig. 2 *D* and *E*), which is carried by KCNQ4 channels (Kubish *et al*. 1999) and which is a characteristic of mature OHCs (Marcotti & Kros, 1999). The size of *I*_k,n_, measured as the difference between the peak and steady state of the deactivating inward current at −124 mV from the holding potential of −84 mV, was 582 ± 37 pA (*n* = 12) in *gata3^+/+^* and 669 ± 76 pA (*n* = 7, *P = *0.2600) *gata3^+/−^* OHCs. The sizes of mature OHCs, measured by cell membrane capacitance (*C*_m_: *gata3^+/+^* 10.5 ± 0.4 pF, *n* = 12; *gata3^+/−^* 10.6 ± 0.4 pF, *n* = 9) and their resting membrane potentials (*V*_m_: *gata3^+/+^* −67 ± 3 mV, *n* = 4; *gata3^+/−^* −70 ± 3 mV, *n* = 4) were similar between the two genotypes. These results suggest that whilst *gata3* haploinsufficiency affects the number or survival of OHCs, it does not affect the biophysical differentiation of surviving cells.

**Figure 3:**
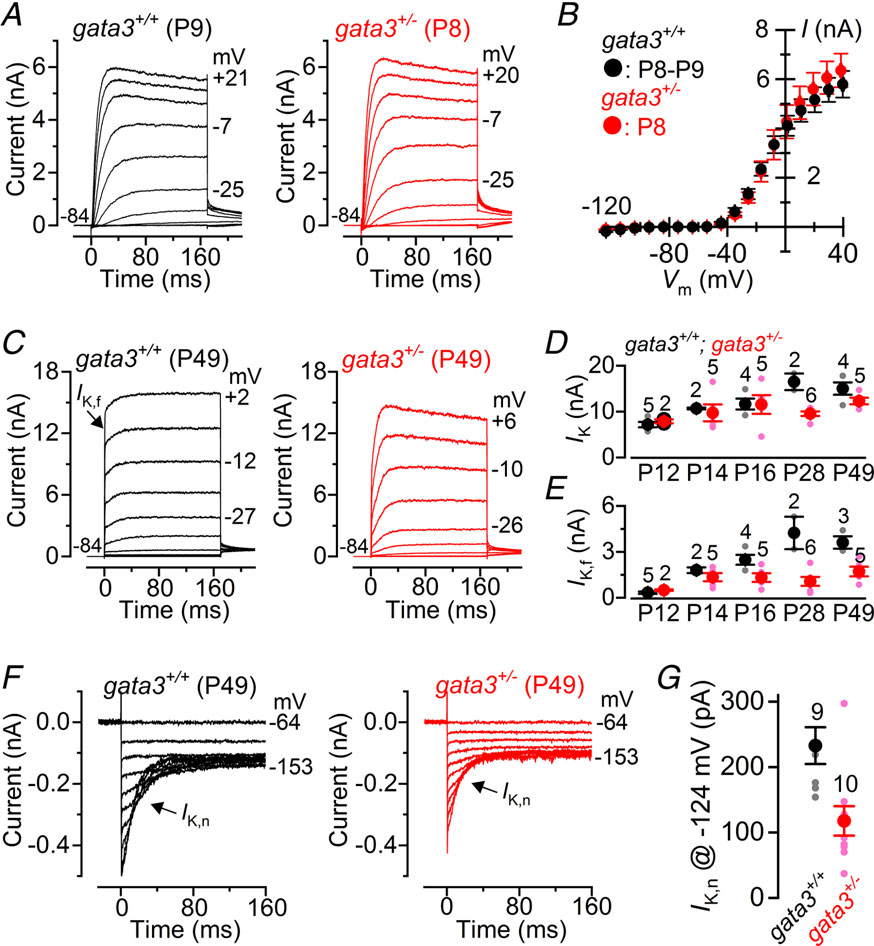
IHCs from *gata3^+/^^−^* mice do not fully differentiate into mature sensory receptors *A*, K^+^ currents elicited from P8 IHCs (control: black; mutant: red) using the same voltage protocol described in Fig. 2 *C*. *B*, current‐voltage curves measured at 160 ms from the voltage‐step onset from IHCs of *gata3^+/+^* (P8–P9: *n* = 4) and *gata3^+/−^* (P8: *n* = 6) mice. *C*, K^+^ currents recorded from adult P49 control and mutant IHCs elicited by 10 mV depolarizing voltage steps from –84 mV. *D* and *E*, size of the total K^+^ current (*I*_K_) measured at 0 mV and 160 ms from the start of the voltage step (*D*) and the isolated *I*_K,f_ (*E*) as a function of postnatal (P) age. *F*, *I*_K,n_ recorded from a *gata3^+/+^* and a *gata3^+/−^* IHC, while applying hyperpolarizing voltage steps in 10 mV increments from the holding potential of –64 mV. Note the characteristic deactivating tail currents (arrows). *G*, size of *I*_K,n_ measured at the membrane potential of –124 mV. Number of IHCs recorded from panels *D, E* and *G* are shown above each column.

In similar studies we found that immature IHCs exhibit comparable current profiles between the two genotypes (Fig. 3 *A* and *B*: *I*_K_: *gata3^+/+^* 3.99 ± 0.44 nA, *n* = 4; *gata3^+/−^* 4.16 ± 0.72 nA, *n* = 6, measured at 0 mV, *P = *0.8614). However, in adult IHCs at P49, the onset of the total K^+^ current in *gata3^+/−^* mice was slower than that of littermate controls (Fig. 3 *C*), suggesting reduced expression of the rapidly activating, large conductance Ca^2+^‐activated K^+^ current *I*_K,f_, which is normally expressed from P12 onwards (Kros *et al*. 1998). The size of the steady‐state total K^+^ current (*I*_K_), measured at 0 mV, was comparable between the two genotypes from P12 to P49, although slightly reduced in *gata3^+/−^* IHCs (two‐way ANOVA: *P = *0.0403) (Fig. 3 *D*). Furthermore, the size of the isolated *I*_K,f_, which we measured at –25 mV and at 2.0 ms after the start of the voltage step, was not significantly different during the first few days after the onset of hearing (Fig. 3 *E*: P12–P14). However, whilst *I*_K,f_ continued to grow with age in control IHCs, it remained the same in *gata3^+/−^* IHCs from P14 onwards (*gata3^+/−^*: one‐way ANOVA: *P = *0.2515). Thus in adult IHCs *I*_K,f_ was significantly smaller than expected (two‐way ANOVA: *P* < 0.0001: Fig. 3 *E*). Mature IHCs, like OHCs, also express the K^+^ current *I*_K,n,_ carried by KCNQ4 channels. We isolated *I*_K,n_ as described above but from a holding potential of −64 mV and found that it was significantly reduced in *gata3^+/−^* IHCs (*P* < 0.0050: Fig. 3 *F* and *G*). Despite the reduced *I*_K,n_ sizes, the resting membrane potentials (*V*_m_: *gata3^+/+^* −70.9 ± 0.9 mV, *n* = 9; *gata3^+/−^* −71.0 ± 0.92 mV, *n* = 9; *P* = 0.84) were not significantly different between the two genotypes. This could be linked to the fact that the immature current profile present in *gata3^+/−^* IHCs could include the inward rectifier *I*_K1_, which contributes to the *V*_m_ in pre‐hearing cells (Marcotti et al., 1999). In addition to preventing the normal biophysical maturation of the IHC basolateral membrane, the lower expression level of gata3 (*gata3^+/−^*) led to a significantly smaller cell size (*C*_m_: 7.5 ± 0.3 pF, *n* = 22, P12–P49) compared to littermate control cells (*C*_m_: 9.6 ± 0.3 pF, *n* = 21, *P* < 0.0001, Student's *t* test). These results show that although the IHC numbers are maintained in *gata3^+/−^* mice, their biophysical differentiation is incomplete and could contribute to the observed hearing loss both in the mouse model and in HDR syndrome. They also show that *gata3* haploinsufficiency differentially affects OHCs and IHCs, suggesting that gata3 function is different in the two cell types.

### *Gata3* is essential for the functional maturation of IHCs

Our measurements from *gata3^+/−^* mice could reflect indirect effects of haploinsufficiency in other cells or tissues, particularly as one identified target of *gata3* is the fibroblast growth factor fgf10, which is critical for cochlear development (Lillevali *et al*. 2006). To target *gata3* deletion primarily to IHCs, we crossed *gata3^fl/fl^* mice with *otof*‐cre^+/−^ mice, in which cre‐recombinase was driven by the hair cell specific *otoferlin* promoter (Yasunaga *et al*. 1999). Otoferlin is normally expressed during embryonic stages E16–E18 and the expression of *cre‐recombinase* is well established by postnatal stage P3 in ∼90% of IHCs and only a small sub‐population of OHCs (Kazmierczak *et al*. 2017).

**Figure 4:**
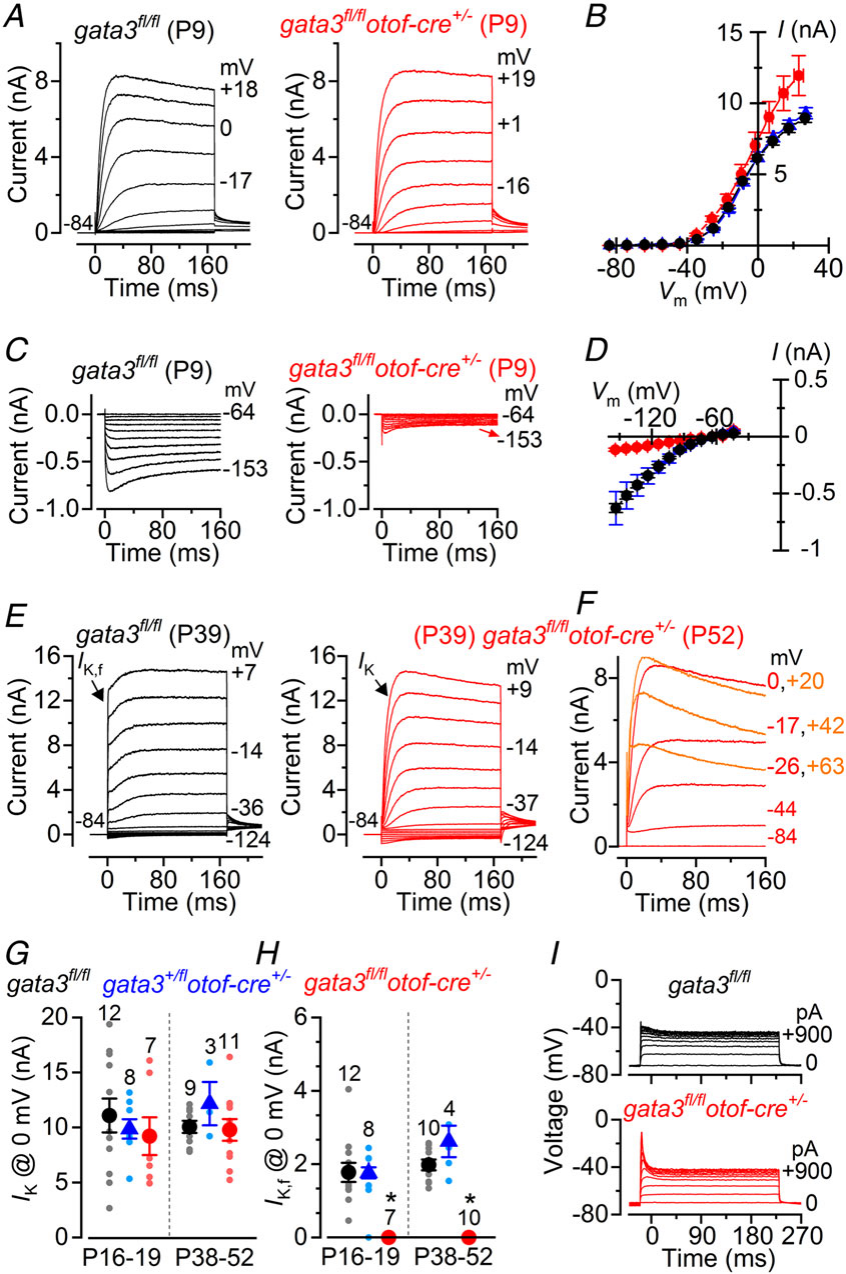
IHCs from *gata3^fl/fl^otof‐cre^+/^^−^* mice retain an immature basolateral membrane current profile *A*, outward K^+^ currents recorded from P9 *gata3^fl/fl^* (control) and *gata3^fl/fl^otof‐cre^+/−^* (gata3 null) IHCs; voltage protocol as described in Fig. 2 *C*. *B*, current‐voltage curves measured at 160 ms from the voltage‐step onset from IHCs of *gata3^fl/fl^* (black: P9–P10: *n* = 19), *gata3^+/fl^otof‐cre^+/−^* (blue: P10: *n* = 3) and *gata3^fl/fl^otof‐cre^+/−^* (red: P9: *n* = 3) mice. *C*, inward K^+^ currents (*I*_K1_) elicited from P9 IHCs using hyperpolarizing voltage steps in 10 mV nominal increments from –64 mV. *D*, steady‐state *I*_K1_‐voltage curves measured at 160 ms from the onset of the voltage step applied to IHCs from control *gata3^fl/fl^* (black), *gata3^+/fl^otof‐cre^+/−^* (blue) and *gata3^fl/fl^otof‐cre^+/−^* (red) mice; number of cells as in panel *B*. *E*, K^+^ currents from adult P39 IHCs elicited by 10 mV hyperpolarizing and depolarizing voltage steps from –84 mV. *F*, example of K^+^ currents recorded from a P52 *gata3^fl/fl^otof‐cre^+/−^* IHC showing evidence for the expression of SK2 channels, which are normally expressed only in immature cells; voltage protocol as in panel *E*. *G* and *H*, size of the total K^+^ current (*G*) and isolated *I*_K,f_ (*H*) recorded from IHCs of all three genotypes just after the onset of hearing (P16–P19) and at adult (P38–P52) stages. *I*, voltage responses recorded from IHCs elicited by applying depolarizing current injections from their respective membrane potentials.

In immature *gata3* floxed mice we found that the size of the outward *I*_K_ current was similar between control IHCs (*gata3^fl/fl^* 6.2 ± 0.3 nA, *n* = 19, measured at 0 mV), heterozygous null IHCs (*gata3^+/fl^otof‐cre^+/−^* 6.2 ± 0.4 nA, *n* = 3) and homozygous null IHCs (*gata3^fl/fl^otof‐cre^+/−^* 7.6 ± 1.3 nA, *n* = 3: overall one‐way ANOVA: *P = *0.999: Fig. 4 *A* and *B*). However, the inward current *I*_K1_ was significantly smaller in *gata3^fl/fl^otof‐cre^+/−^* IHCs (−82 ± 12 pA, *n* = 3, overall one‐way ANOVA: *P* < 0.0001) than it was in either *gata3^fl/fl^* (−322 ± 14 pA, *n* = 19, measured at −124 mV) or *gata3^+/fl^otof‐cre^+/−^* IHCs (−345 ± 71 pA, *n* = 3: Fig. 4 *C* and *D*).

In adult *gata3^fl/fl^otof‐cre^+/−^* IHCs the onset of the K^+^ current was much slower than that recorded in *gata3^fl/fl^* IHCs (Fig. 4 *E*). Moreover, the size of the K^+^ current in 6 out of 11 *gata3^fl/fl^otof‐cre^+/−^* IHCs (P38–P52) was smaller for larger membrane depolarizations (Fig. 4 *F*). This current profile was consistent with the expression of the Ca^2+^‐dependent outward K^+^ conductance. Note that the BK current was almost absent in *gata3^fl/fl^otof‐cre^+/−^* IHCs (Fig. 4 *E*) (Marcotti *et al*. 2004a), suggesting that what we observed was the immature SK2 current (Marcotti *et al*. 2004b). The overall amplitude of the total outward K^+^ current was similar among the different genotypes (Fig. 4 *G*), as was observed in *gata3^+/−^* mice (Fig. 3 *C–E*). The slower activation time course of the K^+^ currents in *gata3^fl/fl^otof‐cre^+/−^* IHCs compared to that of both *gata3^fl/fl^* (Fig. 4 *E*) and *gata3^+/fl^otof‐cre^+/−^* IHCs (data not shown) was due to the absence of the fast activating *I*_K,f_ (Fig. 4 *H*). Consistent with these data, *gata3^fl/fl^otof‐cre^+/−^* IHCs responded to depolarizing current injections with much larger voltage responses than *gata3^fl/fl^* IHCs (Fig. 4 *I*). The immature current profile of *gata3^fl/fl^otof‐cre^+/−^* IHCs was also associated with their significantly smaller size at both P16–P19 (*C*_m_: 7.0 ± 0.9 pF, *n* = 8) and P38–P52 (*C*_m_: 7.7 ± 0.5 pF, *n* = 11) compared to controls at both age ranges (P16–P19: 9.6 ± 0.3 pF, *n* = 12, *P* < 0.05; P38–P52: 11.9 ± 0.8 pF, *n* = 11, *P* < 0.001, Tukey's *post hoc* test from one‐way ANOVA). The size of IHCs between P16–P19 and P38–P52 *gata3^fl/fl^otof‐cre^+/−^* mice was similar (*P = *0.431) and equivalent to that in immature cells at the early stages of postnatal development (Marcotti *et al*. 2003 *a*). Thus gata3 appears to facilitate continued maturation of IHCs in the 2 weeks prior to the onset of hearing. However, the resting membrane potentials were similar between the different genotypes (*gata3^fl/fl^*: ‐68.9 ± 1.6 mV, *n* = 11; *gata3^+/fl^otof‐cre^+/−^*: −72.5 ± 1.7 mV, *n* = 6; *gata3^fl/fl^otof‐cre^+/−^*: −71.1 ± 2.7 mV, *n* = 12; *P = *0.5827), indicating that the K^+^ current active at the resting membrane potential *V*_m_ (*I*_K,n_) was comparable among the different genotypes.

**Figure 5:**
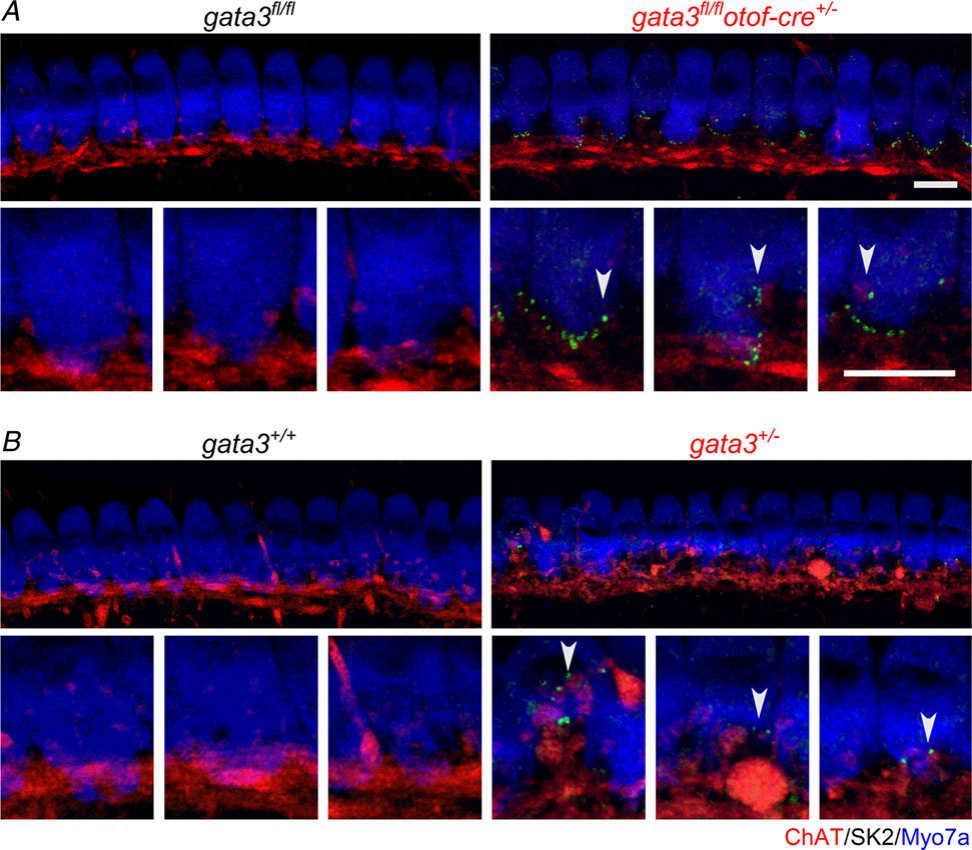
Efferent innervation is retained in IHCs from adult *gata3* deficient mice *A* and *B*, maximum intensity projections of confocal *z*‐stacks taken from the apical cochlear region of control (left columns: *A*, *gata3^fl/fl^*, P29; *B*, *gata3^+/+^*, P49; *n* = 4 mice in each) and *gata3* deficient mice (right columns: *A*, *gata3^fl/fl^otof‐cre^+/−^*, P29; *B*, *gata3^+/−^*, P49; *n* = 4 mice in each) using antibodies against SK2 (green) and ChAT (red). Each panel represents a different mouse. Lower panels show the IHC synaptic region at a higher magnification. Note that the immature‐type SK2 channels are still expressed in the adult *gata3^+/−^* and *gata3^fl/fl^otof‐cre^+/−^* IHCs. Myosin 7a (Myo7a, blue) was used as a hair cell marker. Scale bars 10 μm.

As mentioned above, the K^+^ current profile of adult *gata3^fl/fl^otof‐cre^+/−^* IHCs indicates the presence of a Ca^2+^‐dependent outward K^+^ current (Fig. 4 *F*), which is reminiscent of the current carried by the SK2 channels. This current is normally expressed only transiently in IHCs during pre‐hearing stages of development (Glowatzki & Fuchs, 2000; Katz *et al*. 2004; Marcotti *et al*. 2004b) and in the adult cochlea it is only expressed in OHCs (Oliver *et al*. 2000). The SK2 channels are normally coupled to α9α10 nicotinic acetylcholine receptors (α9α10nAChRs), which form the postsynaptic components of the efferent inhibitory olivocochlear efferent fibres (Simmons *et al*. 1996). We investigated whether the efferent innervation was retained in adult IHCs from *gata3^fl/fl^otof‐cre^+/−^* mice by immunolabelling with the choline acetyl transferase (ChAT) antibody, which labels the presynaptic efferent terminals and with the postsynaptic SK2 antibody. We found that juxtaposed SK2 channels and ChAT‐immunoreactivity were still present in adult IHCs from both in *gata3^fl/fl^otof‐cre^+/−^* (Fig. 5 *A*) and *gata3* ^+/−^ mice (Fig. 5 *B*), but not in the respective controls (Fig. 5), further supporting the interpretation that when gata3 is either reduced or absent IHCs remain immature, thus failing to become fully functional sensory receptors.

**Figure 6:**
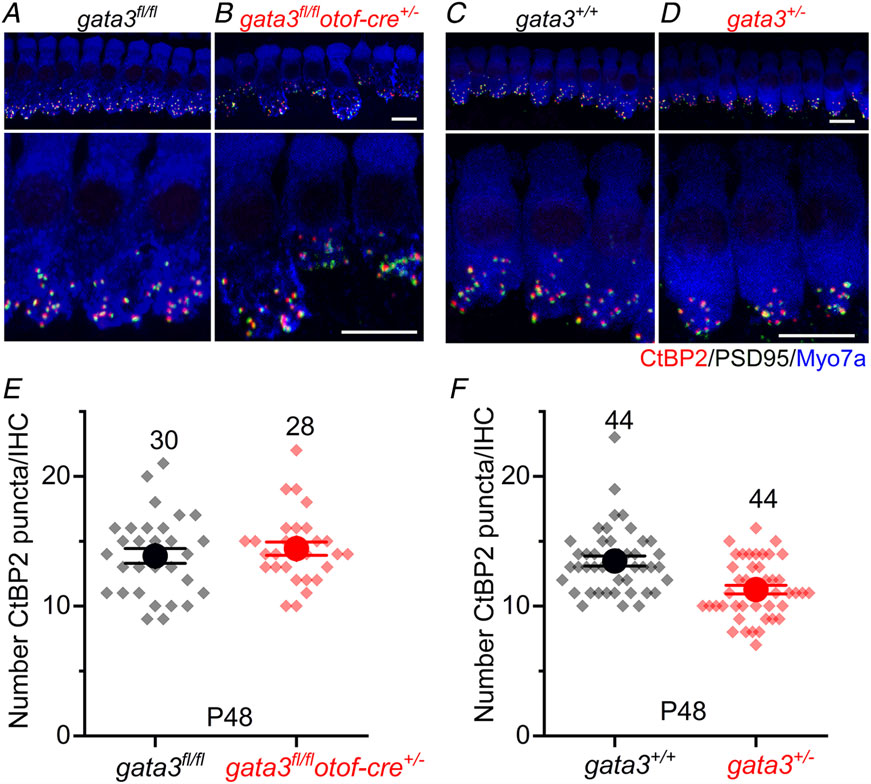
Ribbon synapse number was affected only in IHCs from *gata3^+/^^−^* mice *A–D*, maximum intensity projections of confocal *z*‐stacks taken from the apical cochlear region of control (*A*, *gata3^fl/fl^*; *C*, *gata3^+/+^*) and either *gata3^fl/fl^otof‐cre^+/−^* (*B*) or *gata3^+/−^* (*D*) mice using antibodies against CtBP2 (ribbon synaptic marker: red) and PSD95 (postsynaptic density marker: green). Myosin 7a (Myo7a, blue) was used as a hair cell marker. Scale bars 10 μm. *E* and *F*, number of CtBP2 puncta in the *gata3^fl/fl^otof‐cre^+/−^* (*E*) and *gata3^+/−^* (*F*) mice at P48. Note that in addition to the mean values (large circles), the individual IHC counts are also shown (smaller symbols). In *E*, the ribbon number in *gata3^fl/fl^otof‐cre^+/−^* cells (14.4 ± 2.7, *n = *28 IHCs, 3 mice, *P = *0.468) was no different to that in control *gata3^fl/fl^* IHCs (13.9 ± 3.1, *n = *30 IHCs, 3 mice). In IHCs from *gata3^+/−^* mice (*F*) the number of CtBP2 puncta (11.3 ± 2.2, *n = *44 IHCs, 4 mice) was significantly reduced compared to control cells (13.5 ± 2.5, *n = *44 IHCs, 4 mice, *P *< 0.0006). Averages are means ± SEM.

*Gata3* regulates the development of SGNs and in its absence the postsynaptic densities (PSD) fail to develop, which has an indirect impact on the number of IHC ribbon synapses (Appler *et al*. 2013; Yu *et al*. 2013). Therefore, we evaluated the adult IHC afferent innervation in our mouse models by using antibodies to label afferent ribbon synapses via the presynaptic ribbon protein RIBEYE (CtBP2: red) and PSDs via the protein PSD95 (green). We found that CtBP2 and PSD95 puncta co‐localized at the IHC presynaptic active zones in control mice (*gata3^fl/fl^* and *gata3^+/+^*: Fig. 6 *A* and *C*), *gata3^fl/fl^otof‐cre^+/−^*mice (Fig. 6 *B*) and *gata3^+/−^* mice (Fig. 6 *D*). While, the number of CtBP2 puncta in IHCs from *gata3^fl/fl^otof‐cre^+/−^* adult mice was similar to that of control cells (Fig. 6 *E*, *P = *0.468), in *gata3^+/−^* adult mice it was significantly reduced compared to controls (*P < *0.0006, Student's *t* test, Fig. 6 *F*). We conclude that the number of synaptic ribbons is unlikely to be regulated by the level of *gata3* in IHCs during early postnatal development.

Whilst our data confirmed the early loss of OHCs described for constitutive *gata3* heterozygous null mice, they revealed earlier functional deficits related to loss of gata3 within IHCs that may explain the associated hearing loss. To test this we carried out audiometric tests on the floxed *gata3* mice.

### *Gata3* deletion targeted to hair cells is associated with progressive hearing loss

**Figure 7:**
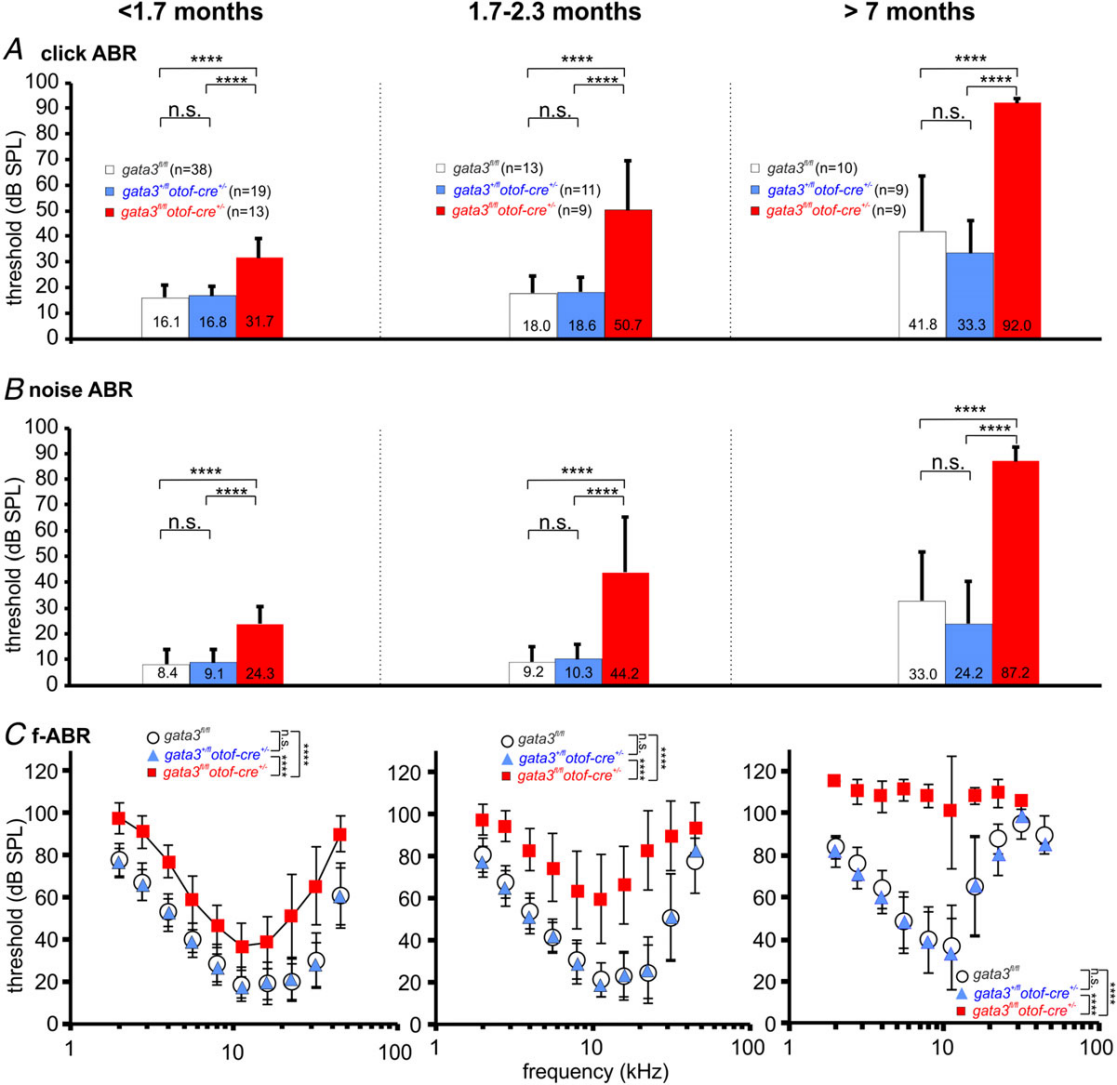
*Gata3^fl/fl^otof‐cre* *^+/−^* knockout mice exhibit a progressive hearing threshold loss *A*, ABR thresholds for click stimuli from control (*gata3^fl/fl^*: white, 10–38 mice; 19–76 ears), heterozygous (*gata3^+/fl^otof‐cre^+/−^*: blue, 9–19 mice; 18–37 ears in each data point) and targeted knockout (*gata3^fl/fl^otof‐cre^+/^* ^−^ red, 9–13 mice; 8–26 ears) mice younger than 1.7 months (left panel), between 1.7 and 2.3 months (middle panel) and older than 7 months (right panel). One‐way ANOVA was *P *< 0.0001 at all ages. *B*, ABR hearing threshold for noise stimuli in *gata3^fl/fl^* (white), *gata3^+/fl^otof‐cre^+/−^* (blue) and *gata3^fl/fl^otof‐cre^+/^* ^−^ (red) at the same ages as in panel *A* (one‐way ANOVA was *P *< 0.0001 at all ages). Number of mice and ears as in panels *A*. *C*, mean ABR thresholds for frequency‐specific pure tone stimulation in *gata3^fl/fl^* (white circles, 10–38 mice; 4–64 ears), *gata3^+/fl^otof‐cre^+/−^* (blue triangles, 9–19 mice; 5–35 ears) and *gata3^fl/fl^otof‐cre^+/^* ^−^animals (red squares, 9–13 mice; 1–26 ears) were found to be significantly raised in knockout mice at all three ages tested (two‐way ANOVA: *P < *0.0001 in all three panels). All values are shown as means ± SD.

**Figure 8:**
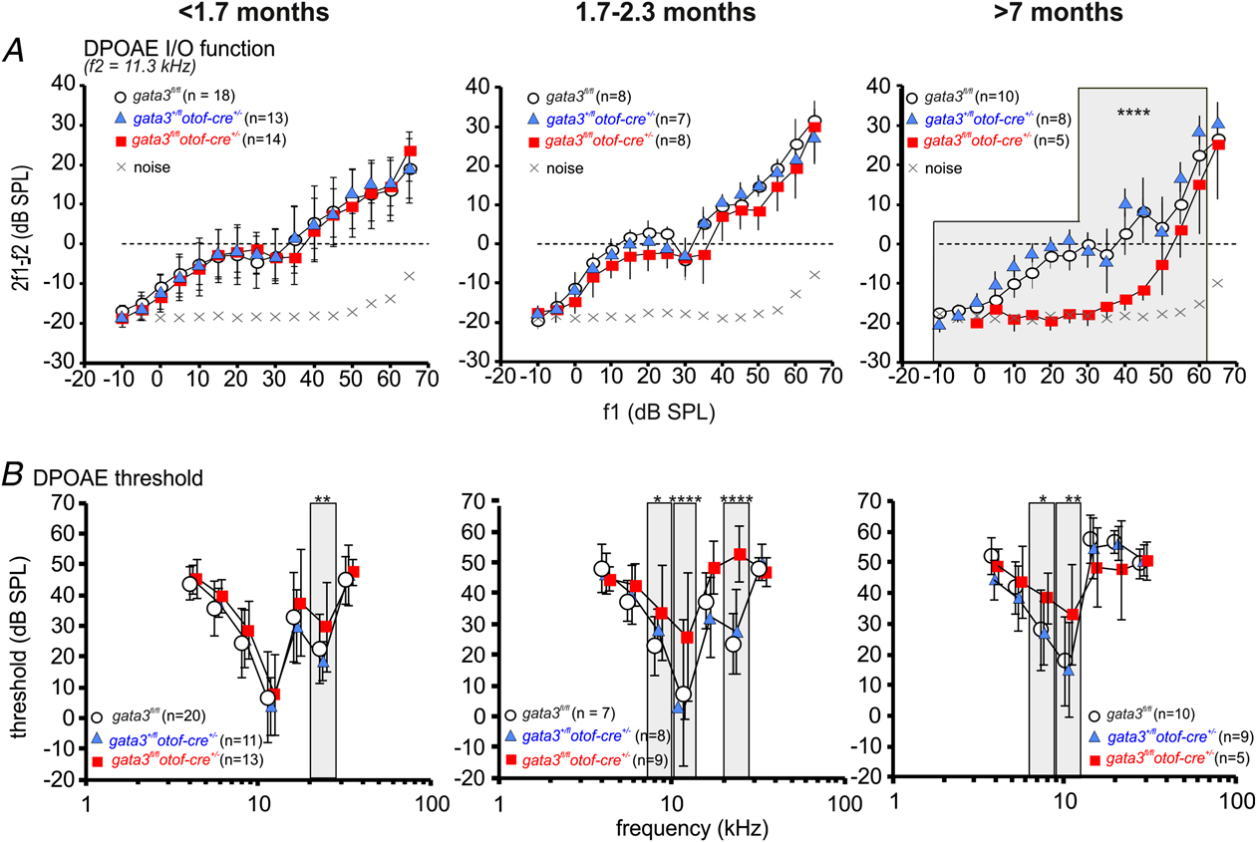
*Gata3^fl/fl^otof‐cre* *^+/−^* knockout mice exhibit deterioration of DPOAEs only at older ages *A*, average DPOAE I/O function from control (*gata3^fl/fl^*: white circles, 8–18 mice, 2–38 ears in each data point), heterozygous (*gata3^+/fl^otof‐cre^+/−^*:blue triangles, 7–13 mice, 2–29 ears) and targeted knockout mice (*gata3^fl/fl^otof‐cre^+/^* ^−^: red squares 5–14 mice, 1–34 ears). Mice younger than 1.7 months (left panel, two‐way ANOVA *P = *0.21); mice between1.7 and 2.3 months (middle panel, *P *< 0.15); mice older than 7 months (right panel, *P *< 0.0001, control and heterorozygous n.s. from *post hoc* test). *B*, mean DPOAE threshold function from *gata3^fl/fl^* (white circles, 8–20 mice; 13–30 ears), *gata3^+/fl^otof‐cre^+/−^* (blue triangles, 8–11 mice; 13–19 ears) and *gata3^fl/fl^otof‐cre^+/^* ^−^mice (red squares, 5–13 mice; 8–18 ears). Mice younger than 1.7 months (left panel, *P *< 0.001); mice between1.7 and 2.3 months (middle panel, *P *< 0.0001); mice older than 7 months (right panel, *P = *0.0856). At all ages, control and heterorozygous were n.s. All values are shown as means ± SD.

We investigated the hearing phenotype in *gata3* conditional knockout mice in which hair cells are preserved, but IHCs fail to become fully mature sensory receptors. Auditory brainstem responses (ABRs), which indicate the activity of afferent auditory neurons downstream of IHCs, were measured in response to clicks (Fig. 7 *A*), noise (Fig. 7 *B*) and pure tones using frequency bands between 4 and 45 kHz with increasing sound pressure level up to 100 dB SPL (Fig. 7 *C*). Before 2 months of age *gata3^fl/fl^otof‐cre^+/−^* mice had significantly higher ABR thresholds for click stimuli (Fig. 7 *A*, *P* < 0.0001, Bonferroni's *post hoc* test from one‐way ANOVA), noise (Fig. 7 *B*, *P* < 0.0001) and pure‐tone stimuli (Fig. 7 *C*, *P* < 0.0001, two‐way ANOVA) compared to *gata3^fl/fl^* and *gata3^+/fl^otof‐cre^+/−^* mice, with the difference increasing with age. ABR thresholds in *gata3^+/fl^otof‐cre^+/−^* mice were similar to those from *gata3^fl/fl^* animals. We measured distortion product otoacoustic emissions (DPOAEs) to investigate whether the increased thresholds in young adult g*ata3^fl/fl^otof‐cre^+/−^* mice (<1.7 months) were due to deterioration of the active cochlear mechanics, as would be expected with loss of OHCs. However, the amplitude of the growth functions (I/O) of DPOAEs (Fig. 8 *A*) and the DPOAE thresholds over a range of frequencies (Fig. 8 *B*) were similar in young adult mice from all three genotypes. Growth function amplitudes and thresholds decayed in older *gata3^fl/fl^otof‐cre^+/−^* mice but the expected increases in DPOAE thresholds were not significantly different between *gata3^fl/fl^* and *gata3^+/fl^otof‐cre^+/−^* mice (*P *> 0.0856, two‐way ANOVA, Fig. 8 *B*). These data indicate that the hearing loss in *gata3^fl/fl^otof‐cre^+/−^* mice is unlikely to be due to OHC loss, at least in young adults.

### Click‐ABR wave Ia and IV amplitude are reduced in *gata3^fl/fl^otof‐cre^+/−^* mice

**Figure 9:**
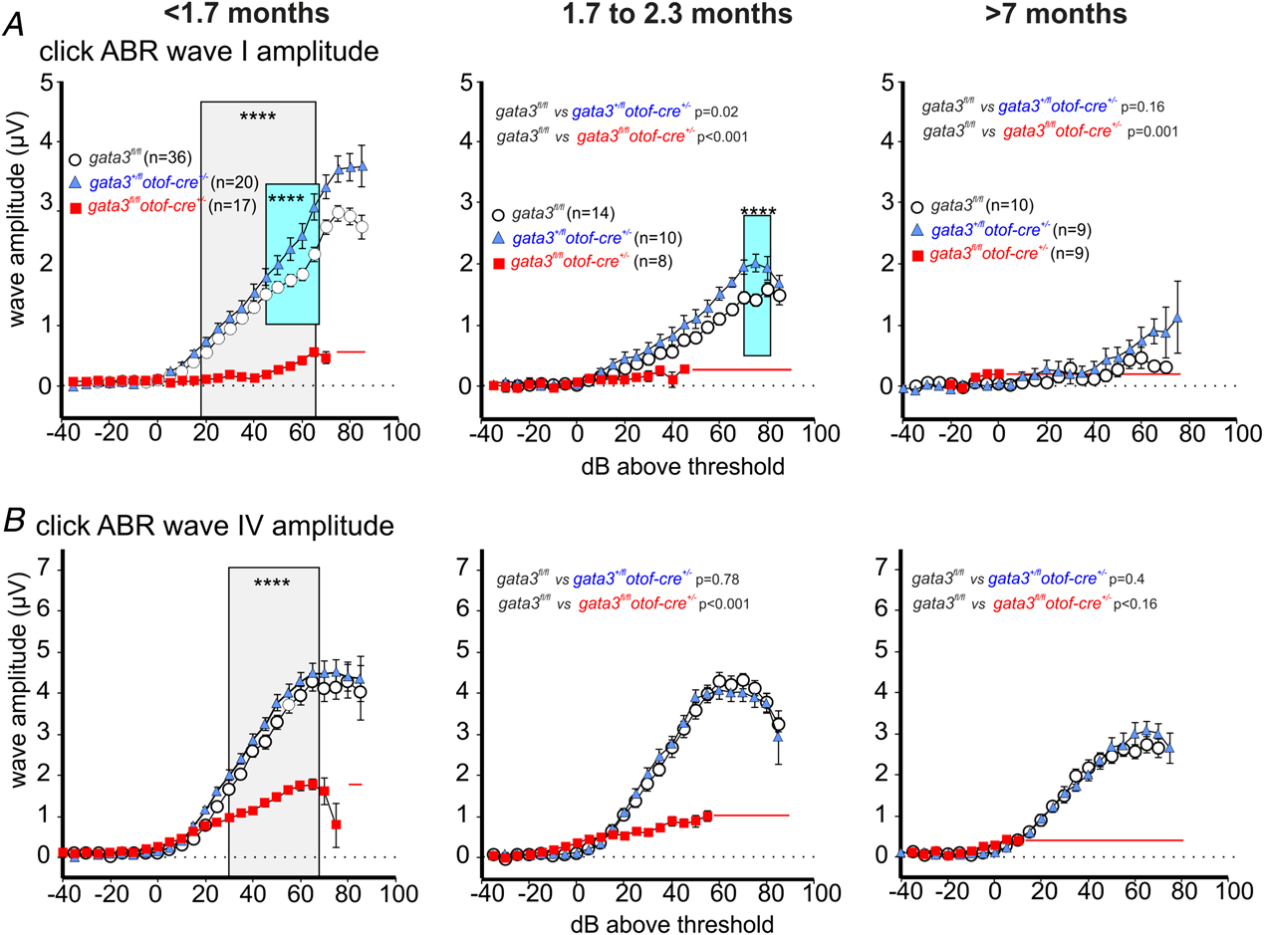
ABR wave amplitudes for low‐ to mid‐frequency click stimuli are reduced in *gata3^fl/fl^otof‐cre* *^+/−^* knockout mice *A*, average ABR wave Ia waveform for click stimulation from control (*gata3^fl/fl^*: white circles, 10–36 mice), heterozygous (*gata3^+/fl^otof‐cre^+/−^*: blue triangles, 9–20 mice) and targeted knockout mice (*gata3^fl/fl^otof‐cre^+/^*: red squares, 8–17 mice). Young adult mice, <1.7 months old, (left panel, repeated‐measurement, RM two‐way ANOVA *P* < 0.0001), mice between 1.7 and 2.3 months (middle panel, control *vs*. heterozygous RM two‐way ANOVA *P* = 0.021, control *vs*. knockout χ^2^ test *P* = 0.0009) and mice older than 7 months (right panel, control *vs*. heterozygous RM two‐way ANOVA *P *= 0.1637, control *vs*. knockout Fisher exact probability test *P* = 0.0013). *B*, average ABR wave IV waveform for click stimulation from control (*gata3^fl/fl^*: white circles, 9–20 mice, 18–35 ears), heterozygous (*gata3^+/fl^otof‐cre^+/−^*: blue triangles, 9–18 mice, 18–31ears) and targeted knockout mice (*gata3^fl/fl^otof‐cre^+/^*: red squares, 7–18 mice, 9–31 ears). Young adult <1.7 months (left panel, RM two‐way ANOVA *P* < 0.0001), mice between 1.7 and 2.3 months (middle panel, control *vs*. heterozygous *P = *0.7779, control *vs*. knockout *P* < 0.0001) and mice older than 7 months (right panel, control *vs*. heterozygous RM two‐way ANOVA *P = *0.4021, control *vs*. knockout Pearson's χ^2^ test *P *< 0.0001). All values are shown as means ± SD.

**Figure 10:**
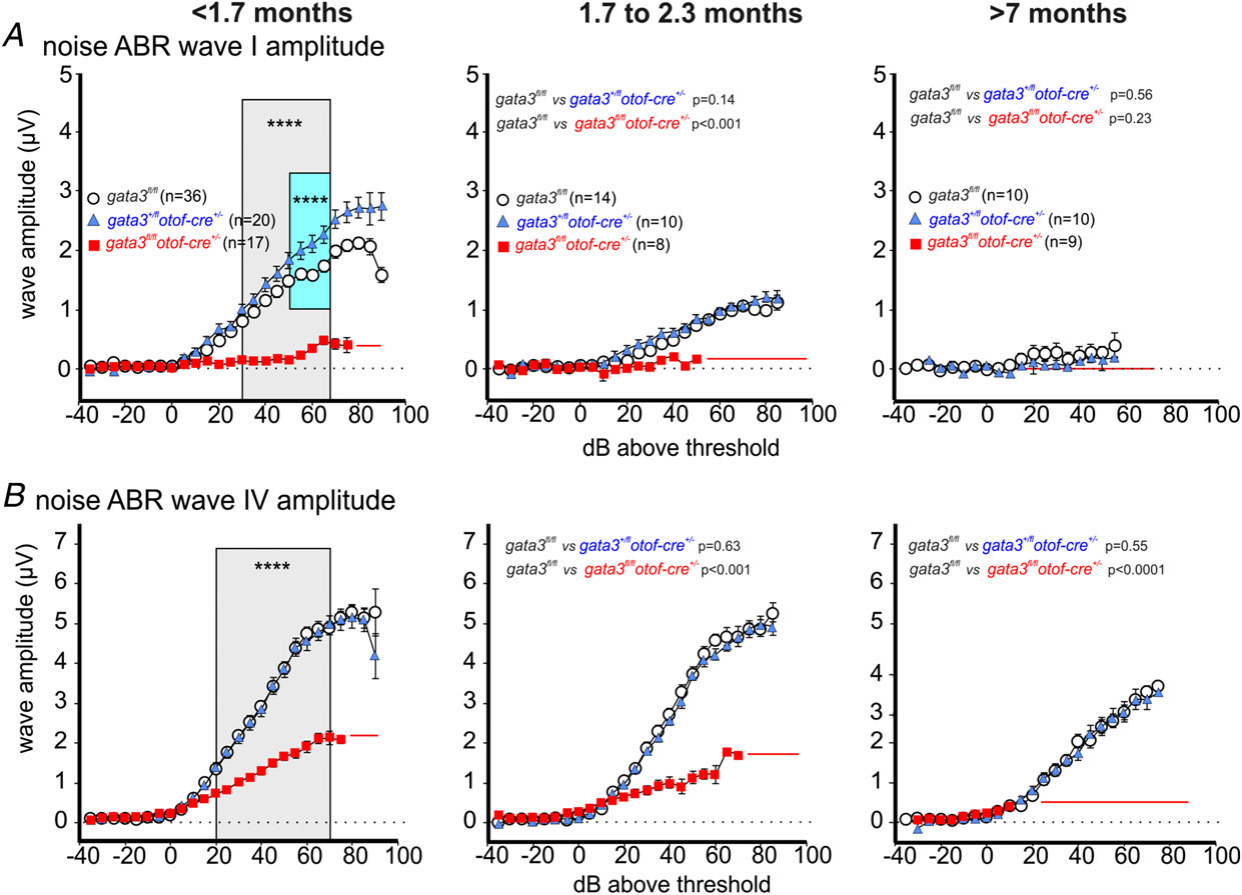
ABR wave amplitudes for high‐frequency noise‐burst stimuli are reduced in *gata3^fl/fl^otof‐cre^+/^^−^*knockout mice *A*, average ABR wave I waveform for noise stimulation from control (*gata3^fl/fl^*: white circles, 10–36 mice), heterozygous (*gata3^+/fl^otof‐cre^+/−^*: blue triangles, 10–20 mice) and targeted knockout mice (*gata3^fl/fl^otof‐cre^+/^*: red squares, 8–17 mice). Young adult mice, <1.7 months old, (left panel, RM two‐way ANOVA *P* < 0.0001), mice between 1.7 and 2.3 months (middle panel, control *vs*. heterozygous RM two‐way ANOVA *P* = 0.1395, control *vs*. knockout Pearson's χ^2^ test *P* = 0.00041) and mice older than 7 months did not show any Ia waveform (control not different from heterozygous, RM two‐way ANOVA *P* = 0.5613. Wave Ia waveforms could be detected in 58, 34 and 13 ears from 35, 18 and 9 mice <1.7 months old; 17, 14 and 2 ears from 17, 14 and 2 mice 1.7–2.3 months old; 4, 2 and 0 ears of 4, 2 and 0 mice >7 months old for control, heterozygote and knockout mice, respectively. *B*, average ABR wave IV waveform for noise stimulation from the same three genotypes and mice listed in panel *A*. Young adult <1.7 months (left panel, RM two‐way ANOVA *P* < 0.0001) mice between 1.7 and 2.3 months (middle panel, control *vs*. heterozygous RM two‐way ANOVA *P = *0.6327, control *vs*. knockout Fisher exact probability test *P = *0.0004) and mice older than 7 months (right panel, control *vs*. heterozygous RM two‐way ANOVA *P* = 0.5465, control *vs*. knockout Pearson's χ^2^ test *P *< 0.0001). Wave VI waveforms could be detected in 59, 34 and 24 ears from 34, 18 and 14 mice <1.7 months old; 27, 19 and 14 ears from 14, 10 and 8 mice 1.7–2.3 months old; 18, 17 and 9 ears of 10, 9 and 7 mice >7 months old for control, heterozygote and knockout mice, respectively. All values are shown as means ± SD

The elevated ABR thresholds (Fig. 7) and the normal DPOAE responses (Fig. 8) in young adult *gata3^fl/fl^otof‐cre^+/−^* mice are consistent with failure in the functional maturation of IHCs (Fig. 4). We investigated supra‐threshold ABR wave amplitudes, which reflect the summed activity of the auditory nerve independent of the contribution from OHCs. Supra‐threshold ABR waveform amplitude analysis was measured as described previously (Moehrle *et al*. 2017). For click stimuli (broadband stimulation centre at 5.4 kHz: Fig. 9) and noise stimuli (broadband stimulation centre at 7.9 kHz, Fig. 10), the amplitudes of ABR waves I and IV declined with age in control and heterozygous mice. This decline was more pronounced for wave I, at the auditory nerve level, than for wave IV, more centrally, which indicates the presence of an intact neural gain. As expected from the similar loss of thresholds for click and noise‐burst stimuli (Figs 9 and 10), the threshold loss and loss of supra‐threshold ABR wave amplitude ranged over the whole frequency range, from low to high frequencies.

## Discussion

We show that *gata3* is crucial for the survival of OHCs as well as for the functional maturation of IHCs at the onset of hearing. Hearing loss due to haploinsufficiency for *gata3* in the *gata3^+/−^* mouse and in human HDR syndrome is probably due to combined defects in both sensory cell types. These new results, together with those previously described in the SGNs (Appler *et al*. 2013; Yu *et al*. 2013), reveal subtle roles for gata3 in different cell types in the coordinated development of the auditory system.

### Gata3 has different functions in OHCs and IHCs

*Gata3* is expressed in hair cells, supporting cells, SGNs and efferent neurons throughout murine embryonic and early postnatal development (Karis *et al*. 2001; van der Wees *et al*. 2004; Lilleväli *et al*. 2006; Luo *et al*. 2013) and protein levels change dynamically with time between these different cell types (Rivolta & Holley 1998; Lawoko‐Kerali *et al*. 2004; Milo *et al*. 2009). At embryonic day E16.5 gata3 is present at higher levels in IHCs and their surrounding non‐sensory cells in the greater epithelial ridge than in OHCs and non‐sensory cells in the lesser epithelial ridge (Milo *et al*. 2009). Adult *gata3^+/−^* mice (>1 month old) suffer general morphological degeneration of cochlear sensory cells, especially of OHCs, in young adults (van der Wees *et al*. 2004). *In vivo* physiological measurements (ABRs and DPOAEs) also point to OHCs as the primary cause for the hearing loss in *gata3^+/−^* mice (van Looij *et al*. 2005). Our data show a significant decrease in OHC numbers in heterozygous null mice shortly after birth, suggesting premature death of OHCs during embryonic development. However, the number of IHCs was normal. In contrast, surviving OHCs matured normally, whilst IHCs failed to acquire the characteristic mature basolateral currents (*I*_K,f_ and *I*_K,n_) and to down‐regulate the immature‐type channels (*I*_SK2_) linked to the cholinergic efferent systems. Thus whilst constitutive haploinsufficiency for *gata3* affects all hair cells, it affects OHCs and IHCs differently. Given the down‐regulation of gata3 in hair cells from E15.5 it is of interest to know if gata3 has an intrinsic function in hair cells from this stage.

### Gata3 has an intrinsic function in the differentiation of IHCs

Otoferlin*‐cre recombinase* is activated from around E16–E18 in the mouse cochlea, with robust activation in the earliest postnatal stages (Kazmierczac *et al*. 2017), by which time IHCs and OHCs are post‐mitotic and morphologically and physiologically defined (Marcotti & Kros, 1999; Marcotti *et al*. 2003). Importantly, the expression of the otoferlin *cre‐recombinase* is evident in ∼90% of IHCs and only a small sub‐population of OHCs (Kazmierczak *et al*. 2017). This allows us to ask whether or not gata3 is functional in differentiating hair cells and if the effects we observed in the *gata3^+/−^* mouse are mediated indirectly by losses of gata3 earlier in development or in other cells or tissues. For IHCs, gata3 appears to have an intrinsic function because in *gata3^fl/fl^otof‐cre^+/−^* mice the phenotype was similar to that in *gata3^+/−^* mice. However, the absence of any effect in *gata3^+/fl^otof‐cre^+/−^* mice suggests less sensitivity to *gata3* haploinsufficiency in IHCs during early postnatal development. Our results predict measurable hearing loss without loss of DPOAEs in *gata3^fl/fl^otof‐cre^+/−^* mice. Indeed, the ABR thresholds were largely elevated (Fig. 7), although they were not as severe as for *gata3^+/−^* mice at a comparable age (van der Wees *et al*. 2004; van Looij *et al*. 2005). At 1.7 months, *gata3^fl/fl^otof‐cre^+/−^* mice had about 20 dB of hearing loss across all frequencies, whereas the *gata3^+/−^* mice had a 30 dB loss (van der Wees *et al*. 2004; van Looij *et al*. 2005). However, full knock‐down of gata3 in IHCs leads to progressive hearing loss, which becomes profound by 7 months (Fig. 7 *C*). Such progression was not observed in *gata3^+/−^* mice, although it might have been masked by the background loss with age for the mouse strain used. Expression of *gata3* is thus necessary in IHCs at least during early adult life as suggested from studies of gata3 deletion at much earlier stages of ear development (Duncan & Fritzsch, 2013).

Evidence for hearing loss linked to lack of function of IHCs was further strengthened through analysis of ABR waves I and IV in *gata3^fl/fl^otof‐cre^+/−^* mice, which reveals severe loss of activity in the auditory nerve and superior olivary complex/lateral lemniscus respectively, at all ages (Figs 9 and 10). The DPOAE measurements (Fig. 8) revealed very little functional decline in OHCs during the first few months, although after 7 months the *gata3^fl/fl^otof‐cre^+/−^* mice have much lower I/O functions, which may reflect some loss of OHCs in cells that lack gata3, consistent with the limited expression of otoferlin cre‐recombinase in OHCs (Kazmierczac *et al*. 2017). We conclude that gata3 has an intrinsic function in the differentiation of IHCs and for successful onset of hearing in mice at P12–P14.

### Gata3 is unlikely to directly regulate the differentiation and survival of OHCs

*Gata3* haploinsufficiency did not compromise the physiological differentiation of OHCs but led to their loss across all frequencies during embryonic development, with the most severe losses in the apical, low frequency cochlear region. Our observation that OHC numbers were maintained in *gata3^fl/fl^otof‐cre^+/−^* mice suggests either that gata3 was not knocked down in the OHCs or that OHC survival depends upon gata3 expression in other cells, notably the surrounding non‐sensory cells. The latter hypothesis is supported by the fact that the number of OHCs and the DPOAEs were unaffected in *gata3^fl/fl^otof‐cre^+/−^* mice, despite some degree of otoferlin cre‐recombination in these cells (Kazmierczac *et al*. 2017). OHC survival is normally dependent on fgf signalling (Pirvola *et al*. 2000; Atkinson *et al*. 2015) and gata3 appears to regulate fgf10 during inner ear development (Lilleväli *et al*. 2006; Economou *et al*. 2013), although this is not the case in SGNs (Appler *et al*. 2013). We conclude that gata3 is not required for the differentiation OHCs and that their loss in *gata3^+/−^* mice could depend on loss of gata3 in adjacent supporting cells.

### HDR syndrome

*Gata3^+/−^* mice provide a reasonable model for the auditory component of HDR syndrome (van Esch *et al*. 2000; Muroya *et al*. 2001; van der Wees *et al*. 2004; van Looij *et al*. 2005; Luo *et al*. 2013; van Beelen *et al*. 2014). The implication of our data is that the associated deafness can be explained largely by the composite effects of incomplete IHC differentiation and poor survival of OHCs. In addition, the increased loss of non‐sensory cells and SGNs with time (van der Wees *et al*. 2004) suggests a more widespread effect of gata3 haploinsufficiency on cell survival. Given the lack of impairment in IHCs in *gata^+/fl^otof‐cre^+/−^* mice, we speculate that much of the impact of haploinsufficiency in HDR syndrome occurs during development. This is consistent with the observation that most of the hearing loss in *gata3^+/−^* mice is evident 1 month after birth and there is little additional loss thereafter on top of the observed age‐related decline (van der Wees *et al*. 2004).

### Coordination of maturation and innervation by gata3 during auditory system development

Targeted deletion of gata3 from mid‐embryonic development disrupts both the timing and the organisation of the peripheral neuronal projections and causes the loss of SGNs by birth (Appler *et al*. 2013; Duncan & Fritzsch, 2013). Gata3 is required for expression of the transcription factor Mafb and in *Mafb* null mice the postsynaptic densities (PSD) in SGNs fail to develop at P6 (Yu *et al*. 2013). This is associated with disruption of presynaptic ribbons and a hearing loss of ∼10 dB across the frequency range (Yu *et al*. 2013). Deletion of *gata3* from IHCs during late embryonic development has no effect on the number or disposition of synaptic ribbons. However, the number of ribbons is significantly lower in *gata3^+/−^* mice (Fig. 5). We know little about the cumulative effects of haploinsufficiency in IHCs but it seems most likely that the lower number of ribbons is a consequence of haploinsufficiency in SGNs. Deletion of *gata3* in SGNs from E13.5 leads to around 50% reduction in IHC ribbons labelled with antibodies to CtBP2 (Yu *et al*. 2013) and we observed a decrease of only 10–15%. Haploinsufficiency affects neurite extension in *gata3^+/−^* mice (Appler *et al*. 2013) and may influence ribbon numbers via failure to develop some PSDs.

Gata3 may influence the timing of differentiation in different cell types. Its developmental expression coincides with a period during which supernumerary hair cells can be induced or derived (Kelley *et al*. 1993; White *et al*. 2006) and it is selectively down‐regulated in hair cells in a basal to apical progression shortly after the expression of the transcription factor Atoh1 (Rivolta & Holley, 1998; Lee *et al*. 2006). It remains higher for longer in IHCs (Milo *et al*. 2009), which reach maturity later than OHCs (Marcotti *et al*. 2003) and its deletion in SGNs leads to premature projection of dendrites (Appler *et al*. 2013). Thus continued expression of critical levels of gata3 could maintain cell competence during development, which is why deletion from IHCs prevents their developmental progression.

## Additional information

### Competing interests

The authors declare no conflict of interest.

### Author contributions

All authors helped with the collection and analysis of the data. W.M. and M.C.H. coordinated the study and wrote the paper. M.C.H. conceived the work. All authors have approved the final version of the manuscript and agree to be accountable for all aspects of the work. All persons designated as authors qualify for authorship and all those who qualify for authorship are listed.

### Funding

This work was supported by Action on Hearing Loss (562:SHE:MH) and The Rosetrees Trust (to M.C.H.), the Dudley and Geoffrey Cox Charitable Trust (to MCH) and the Wellcome Trust (102892 to W.M.) in the UK. In Germany, it was supported by the Deutsche Forschungsgemeinschaft (FOR 729, part projects A2 and 6, 2060 project FE 438/5‐1, KN316/4‐1, KN316/12‐1, SFB 612, project A8), the Hahn Stiftung (Index AG). S.L.J. is a Royal Society URF.
